# Histone demethylase KDM4A plays an oncogenic role in nasopharyngeal carcinoma by promoting cell migration and invasion

**DOI:** 10.1038/s12276-021-00657-0

**Published:** 2021-08-12

**Authors:** Jingyi Zhao, Bingyan Li, Yongxia Ren, Tiansong Liang, Juan Wang, Suna Zhai, Xiqian Zhang, Pengcheng Zhou, Xiangxian Zhang, Yuanyuan Pan, Fangfang Gao, Sulan Zhang, Liming Li, Yongqiang Yang, Xiaoyu Deng, Xiaole Li, Linhui Chen, Daoke Yang, Yingjuan Zheng

**Affiliations:** 1 Radiotherapy Department, the First Affiliated Hospital of Zhengzhou University, Zhengzhou, PR China; 2 Radiotherapy Department, Huaihe Hospital of Henan University, Kaifeng, PR China; 3 Institute of Radiation Therapy and Tumor Critical Care of Zhengzhou University, Zhengzhou, PR China; 4 Henan Key Laboratory of Molecular Radiotherapy, Zhengzhou, PR China

**Keywords:** Oncogenes, Cancer

## Abstract

Compelling evidence has indicated the vital role of lysine-specific demethylase 4 A (KDM4A), hypoxia-inducible factor-1α (HIF1α) and the mechanistic target of rapamycin (mTOR) signaling pathway in nasopharyngeal carcinoma (NPC). Therefore, we aimed to investigate whether KDM4A affects NPC progression by regulating the HIF1α/DDIT4/mTOR signaling pathway. First, NPC and adjacent tissue samples were collected, and KDM4A protein expression was examined by immunohistochemistry. Then, the interactions among KDM4A, HIF1α and DDIT4 were assessed. Gain- and loss-of-function approaches were used to alter KDM4A, HIF1α and DDIT4 expression in NPC cells. The mechanism of KDM4A in NPC was evaluated both in vivo and in vitro via RT-qPCR, Western blot analysis, MTT assay, Transwell assay, flow cytometry and tumor formation experiments. KDM4A, HIF1α, and DDIT4 were highly expressed in NPC tissues and cells. Mechanistically, KDM4A inhibited the enrichment of histone H3 lysine 9 trimethylation (H3K9me3) in the HIF1α promoter region and thus inhibited the methylation of HIF1α to promote HIF1α expression, thus upregulating DDIT4 and activating the mTOR signaling pathway. Overexpression of KDM4A, HIF1α, or DDIT4 or activation of the mTOR signaling pathway promoted SUNE1 cell proliferation, migration, and invasion but inhibited apoptosis. KDM4A silencing blocked the mTOR signaling pathway by inhibiting the HIF1α/DDIT4 axis to inhibit the growth of SUNE1 cells in vivo. Collectively, KDM4A silencing could inhibit NPC progression by blocking the activation of the HIF1α/DDIT4/mTOR signaling pathway by increasing H3K9me3, highlighting a promising therapeutic target for NPC.

## Head and neck cancer: Signaling pathway identified as potential treatment target

Clarifying the mechanism behind the progression of nasopharyngeal carcinoma may hold the key to novel therapies. Lysine specific demethylase 4A (KDM4A) is encoded by a cancer-associated gene and is overexpressed in tumors including human nasopharyngeal carcinoma. However, how the KDM4A enzyme influences NPC progression is unclear. Daoke Yang and Yingjuan Zheng at the First Affiliated Hospital of Zhengzhou University, Zhengzhou, China, and co-workers examined KDM4A in human NPC tumor samples and in experiments on mice. They found that increased expression levels of two proteins, HIF1α and DDIT4, directly correlated with high levels of KDM4A. The overexpression of KDM4A promotes expression of HIFα, which increases DDIT4 levels, activating a signaling pathway that promotes cancer cell proliferation, migration and invasion. This pathway may provide a therapeutic target for NPC.

## Introduction

Human nasopharyngeal carcinoma (NPC) is a common head and neck malignancy and the common primary malignant tumor arising in the nasopharynx; it has an especially high incidence in Southeast Asia and South China^1,2^. Additionally, the etiology of NPC is unclear, but the disease is related to Epstein-Barr virus and human papilloma virus infections, and immune factors also play a role in its carcinogenesis^3^. Although advanced treatment strategies for NPC have been developed in recent years, obtaining effective control of NPC is still a major clinical challenge^4^. Additionally, available treatments for NPC are limited by a variety of serious side effects, and surgical treatment is rarely appropriate^5^. Thus, analysis of the underlying molecular mechanisms of NPC is urgently needed to provide new insight for improving NPC treatment.

Histone posttranslational modification is an important type of epigenetic modification, and the methylation of lysine residues has been widely studied^6^. Histone demethylase jumonji C domain 2 A (JMJD2A), also known as lysine-specific demethylase 4 A (KDM4A), is a potential oncogene and is highly expressed in human tumors^7^. Indeed, it has been reported that KDM4A plays an important role in the tumorigenesis and progression of NPC, thus indicating KDM4A as a promising biomarker and target for the treatment of NPC^8^. Importantly, KDM4A silencing promotes the accumulation of histone 3 lysine 9 trimethylation (H3K9me3) at the sites of hypoxia-inducible factor-1α (HIF1α), leading to decreased HIF1α mRNA expression and stability^9^.

Transcription factors typically regulate gene expression via their roles at DNA binding sites^10^. In addition, transcription factors participate in many human diseases, such as cancers, and account for approximately 20% of all oncogenes verified to date, so they are increasingly being used as potential therapeutic targets in drug development^11,12^. Previous evidence has demonstrated that activation of the transcription factor HIF1α promotes the glycolysis and tumorigenesis of NPC cells^13^. DNA damage-induced transcript 4 (DDIT4) serves as the link between HIF1α and the mechanistic target of rapamycin (mTOR) signaling pathway and regulates the fate of adult stem cells^14^. However, the expression and function of DDIT4 in NPC have not been reported. The microRNA (miR)-99a/mTOR axis provides new insights into the pathogenesis of NPC and represents a potential therapeutic target for NPC^15^. It has also been demonstrated that activation of the phosphoinositide 3-kinase (PI3K)/mTOR signaling pathway is associated with a poor prognosis in NPC^16^.

Based on the above evidence, we hypothesized that the KDM4A/HIF1α/DDIT4/mTOR axis might be involved in the development of NPC and therefore investigated its underlying regulatory mechanism, with the aim of identifying new therapeutic targets for the treatment of NPC.

## Materials and methods

### Ethics statement

The experiment was approved by the Ethics Committee of the First Affiliated Hospital of Zhengzhou University and conducted in compliance with the *Declaration of Helsinki*. All participants signed informed written consent forms. The experiments involving animals were performed in line with the Guide for the Care and Use of Laboratory Animals issued by US National Institutes of Health and strictly adhered to the principle of minimizing pain, suffering and discomfort to experimental animals.

### Study subjects

For this study, 55 pairs of NPC tissues and adjacent tissues (nontumor tissues > 5 cm away from the tumor’s outer margin taken from the same patient, control group) were collected from NPC patients undergoing surgery at the First Affiliated Hospital of Zhengzhou University from January 2011 to October 2013. There were 37 males and 18 females, aged from 24 to 70 years, with an average age of 48 years. The included NPC patients received no radiotherapy, chemotherapy or other treatment before surgery. Diagnosis was confirmed by two experienced pathologists. The tissues were divided into two parts: one portion was immediately stored in liquid nitrogen, and the other was fixed with 10% formaldehyde, paraffin-embedded and sectioned. The patients were followed up for 60 months, and survival analysis was performed using the Kaplan–Meier method. During the follow-up period, the patient’s death was taken as the endpoint event. If no event occurred, the last follow-up time was regarded as the endpoint. The interval between the date of the patient’s operation and the date of death was defined as overall survival (OS).

### Immunohistochemistry

Paraffin specimens were collected, sectioned, and dewaxed, followed by routine immunohistochemical staining. The primary antibody rabbit anti-KDM4A (ab191433, 1: 250) and secondary antibody immunoglobulin G (IgG) (ab150083, 1:100) were purchased from Abcam Inc. (Cambridge, UK). Tissues treated with normal saline instead of the primary antibody were used as a negative control (NC). To judge the staining results, the number of positively stained cells was counted in five randomly selected lesions under a microscope. The density of positive cells was also semiquantitatively graded according to the percentage of positive cells as follows: < 15% positive cells, (-); 15–25% positive cells, (+); 25–50% positive cells, (++); 50–75% positive cells, (+++), and > 75% positive cells, (++++).

### Cell culture

Four NPC cell lines, SUNE1, SUNE-2, 5–8 F, and 6–10B; one control cell line, the NPC epithelial cell line NP69; and embryonic kidney cells (HEK-293T), were purchased from Zhen’ao Bio (Guangzhou, China). NP69 cells were cultured in keratinocyte/serum-free medium (Invitrogen, Carlsbad, CA, USA) supplemented with epidermal growth factor (EGF). All other NPC cells were resuspended and cultured in Roswell Park Memorial Institute (RPMI) 1640 medium (Gibco, Rockville, MD, USA) supplemented with 10% fetal bovine serum (FBS, 26140079, Gibco, Cal, USA) and 1% penicillin and streptomycin solution in a 5% CO_2_, 37 °C incubator (BB15, Thermo Fisher Scientific Inc., Waltham, MA, USA) with saturated humidity. The culture medium was changed every 24 h, and the cells were passaged every 72 h. The culture medium was removed, and the cells were washed twice with phosphate-buffered saline (PBS) and digested with 0.25% trypsin for 3 min. The digestion was terminated by adding RPMI 1640 medium containing 10% FBS, and the cells were dispersed into a single-cell suspension with a pipette. After routine passaging, cells in the logarithmic growth phase were used to detect KDM4A expression using reverse transcription quantitative polymerase chain reaction (RT-qPCR) and Western blot analysis. The cell with the highest KDM4A expression was selected for subsequent experiments.

### Cell transfection and grouping

When the confluence of SUNE1 cells reached 80–90%, transfection was performed according to the instructions for Lipofectamine 2000 transfection reagents (11668-019, Invitrogen, New York, California, USA). SUNE1 cells were treated with small interfering RNA (si)-KDM4A plasmid, si-NC plasmid, histone demethylase KDM4A inhibitor (JIB-04, 2 µM, for 24 h), the vehicle for JIB-04 (dimethyl sulfoxide, DMSO), si-NC + cycloheximide (CHX), and si-KDM4A + CHX. In addition, SUNE1 cells were transfected with si-NC + overexpression (OE)-NC, si-KDM4A + OE-NC, si-NC + OE-HIF1α, or si-KDM4A + OE-HIF1α plasmids. Moreover, SUNE1 cells were transfected with OE-NC + si-D-NC (NC for si-DDIT4), OE-HIF1α, OE-NC + si-DDIT4, or OE-HIF1α + si-DDIT4 plasmids. Additionally, SUNE1 cells were treated with OE-D-NC (NC for OE-DDIT4) + PBS, OE-DDIT4 + PBS, OE-D-NC + mTOR pathway inhibitor everolimus, or OE-DDIT4 + everolimus. Finally, SUNE1 cells were treated with OE-K-NC (NC for OE-KDM4A) + PBS, OE-KDM4A + PBS, OE-K-NC + everolimus, or OE-KDM4A + everolimus. All the above plasmids were purchased from Ribobio Biological (Guangzhou, China).

### RT-qPCR

Tissue homogenate or 100 μL cells was placed in a reaction tube, and 1 mL of TRIzol (15596-018, Beijing Solarbio Science & Technology Co., Ltd., Beijing, China) was added to extract total RNA. RNA (2 µg) was synthesized into complementary DNA (cDNA) at 42 °C for 50 min by TaqMan reverse transcription reagent (purchased from RNPChe), and PCR (50 μL reaction system) was used to amplify the target gene fragment. The primers used in PCR were all synthesized by Sigma-Aldrich Chemical Company (St Louis, MO, USA) (Supplementary Table 1). Next, 2 µg of total cDNA was used as a template, and glyceraldehyde-3-phosphate dehydrogenase (GAPDH) was used as an internal reference primer. Based on the CT value, the expression of the target gene was analyzed by the 2^-ΔΔCt^ method with the following formulas: △△ Ct = △ Ct experimental group- △ Ct control group, △ Ct = Ct (target gene) - Ct (internal reference); relative transcription level of target gene mRNA = 2^-△△CT^.

### Western blot analysis

Precooled phenylmethylsulfonyl fluoride (PMSF)-containing radioimmunoprecipitation assay (RIPA) lysis buffer (R0010, Solarbio) was used in accordance with the instructions to extract total protein from cells or tissues. The protein concentration of each sample was determined using a bicinchoninic acid (BCA) kit (20201ES76, Yeasen Biotechnology Co., Ltd., Shanghai, China). Quantification was performed according to different concentrations. After the protein was separated by polyacrylamide gel electrophoresis, the protein was transferred onto a polyvinylidene fluoride (PVDF) membrane (Millipore Corp, Bedford, MA, UAS) by a wet transfer method. Following blocking with 5% bovine serum albumin (BSA) at room temperature for 1 h, the membrane was probed with the diluted primary rabbit antibodies against KDM4A (ab191433, 1:5000), H3K9me3 (ab8898, 1:2000), HIF1α (ab2185, 1:1000), DDIT4 (ab191871, 1:1000), mTOR (ab2732, 1:2000), phosphorylated mTOR (ab109268, 1:5000), 4EBP1 (ab2606, 1:3000), phosphorylated 4EBP1 (ab47365, 1:1000), H3 (ab4729, 1:1000), Ki67 (ab92742, 1:5000), cyclin D1 (ab134175, 1:3000), matrix metalloproteinase (MMP)-2 (ab37150, 1:2000), MMP-9 (ab73734, 1:2000), Bcl-2-associated X protein (Bax) (ab32503, 1:2000), B-cell lymphoma 2 (Bcl-2) (ab59348, 1:1000), and GAPDH (ab181602, 1:10000), incubated overnight at 4 °C, and washed 3 times with Tris-buffered saline Tween-20 (TBST) (10 min per wash). Then, the membrane was reprobed with horseradish peroxidase (HRP)-labeled goat anti-rabbit IgG (ab6721, 1:5000) secondary antibody at room temperature for 1 h, washed with TBST 3 times for 10 min, and developed with developing solution. All antibodies were from Abcam. Finally, GAPDH was used as the internal reference, and the relative protein content was expressed as the grayscale value of the corresponding protein band/the grayscale value of the internal protein band detected by Quantity One v4.6.2 software.

### Chromatin immunoprecipitation (ChIP)

A ChIP kit (Millipore Corp, Bedford, MA, UAS) was used to study H3K9me3 enrichment in the HIF1α gene promoter region. Cells in logarithmic growth phase were added to 1% formaldehyde and fixed at room temperature for 10 min to cross-link the DNA and proteins. Next, the cells were disrupted by 15 cycles of ultrasonic treatment consisting of 10 s ultrasound followed by 10 s pauses to break DNA into fragments of appropriate size. Then, the samples were centrifuged at 4 °C and 13000 rpm (some DNA fragments remained as input). The supernatant was collected and divided into three tubes, probed with the NC antibody of normal mouse IgG (ab18413, Abcam) and the target protein-specific antibody (H3K9me3, ab8898, anti-rabbit, Abcam), and incubated at 4 °C overnight. Protein agarose/sepharose was used to precipitate the endogenous DNA-protein complexes. After centrifugation, the supernatant was discarded, and the nonspecific complexes were washed with buffer. After decrosslinking, the DNA fragments were recovered by phenol/chloroform extraction purification. The primers specific for the HIF1α gene promoter region are shown in Supplementary Table 1.

### 3-(4,5-Dimethylthiazol-2-yl)-2,5-diphenyltetrazolium bromide (MTT) assay

The cultured cells were washed twice with PBS solution, detached with 0.25% trypsin and prepared into a single-cell suspension. After counting, the cells were seeded into 96-well plates at a density of 5 × 10^6^ - 6 × 10^6^ cells/well (0.2 mL) with six replicate wells, and then incubated in an incubator. After 24 h, 48 h and 72 h of culture, the plates were removed, and the medium was replaced with medium containing 10% MTT solution (5 g/L) (GD-Y1317, Guduo Biotechnology, Shanghai, China). The culture continued for 4 h, and the supernatant was discarded. Then, 100 μL DMSO (D5879, 100 mL, Sigma-Aldrich) was added to each well, and the plate was gently shaken to mix the samples for 10 min to dissolve the formazan crystals produced by living cells. The optical density (OD) value of each well at 490 nm was detected using a microplate reader (BS-1101, Detie Experimental Equipment Co., Ltd., Nanjing, Jiangsu, China). A cell viability curve was drawn with the time point as the abscissa and the OD value as the ordinate.

### Transwell assay

During cell migration experiments, cells were seeded into Transwell chambers with 8-μm pores at a density of 5 × 10^4^ cells/mL (200 µL). The corresponding cells or 500 μL medium with 10% FBS was inoculated in the lower chamber, with three replicates set for each group; the cells were cultured at 37 °C with 5% CO_2_ for 24 h. Thereafter, the cells were fixed in 4% paraformaldehyde at 4 °C, stained with crystal violet (C0121, Beyotime Biotechnology Co., Shanghai, China) for 20 min and washed twice with PBS. The cells on the surface of the chamber were wiped off with cotton balls and observed under an inverted fluorescence microscope (TE2000, Beijing You Nikon Biotechnology Co., Ltd., Beijing, China) in five randomly selected visual fields, and the mean values were obtained.

For the cell invasion experiments, precooled Matrigel (40111ES08, Yeasen Biotechnology Co., Ltd., Shanghai, China) diluted with serum-free DMEM (Matrigel: DMEM = 1:2) was spread in the upper Transwell chamber (3413, You Nikon) and incubated in a 37 °C incubator for 4-5 h. Next, 100 µL serum-free medium was used to dilute the transfected cells to prepare a cell suspension with a density of 1 × 10^6^ cells/mL. The lower chamber was supplemented with 500 µL DMEM containing 10% FBS, with 3 duplicate wells for each group, and the samples were cultured at 37 °C with 5% CO_2_ for 24 h. The remaining steps were consistent with those of the cell migration experiment.

### Flow cytometry

After transfection for 48 h, the cells were detached by ethylenediamine tetraacetic acid (EDTA)-free 0.25% trypsin, collected and centrifuged, and the supernatant was discarded. According to the instructions of the Annexin-V-fluorescein isothiocyanate (FITC) apoptosis detection kit (Shanghai Shuojia Biotechnology Co., Ltd, Shanghai, China), Annexin-V-FITC, propidium iodide (PI) and 4-(2-hydroxyethyl)-1-piperazineëthanesulfonic acid (HEPES) buffer solution was formulated into annexin-V/PI staining solution at a ratio of 1:2:50. Then, 1 × 10^6^ cells were resuspended in 100 µL staining solution, mixed, and incubated at room temperature for 15 min, and then 15 mL HEPES buffer solution was added. Flow cytometry (Bio-Rad ZE5, BIO-RAD) was used to detect the level of apoptosis. The maximum absorption wavelength of FITC was 488 nm, and the excitation wavelength was 525 nm. The maximum absorption and emission wavelengths of the PI-DNA complex were 535 nm and 615 nm, respectively.

### Tumor formation in nude mice

Five groups of stably transfected cell lines (blank, si-NC + OE-DDIT4, si-KDM4A + OE-DDIT4, OE-KDM4A + PBS, OE-KDM4A + everolimus, OE-KDM4A + DMSO, OE-KDM4A + JIB-04) were prepared. Then, 4-week-old specific pathogen-free (SPF) female nude mice (weighing 20–22 g, *n* = 30) (Shanghai SLAC Laboratory Animal Co., Ltd, Shanghai, China) were prepared for experiments. The nude mice were randomly divided into 5 groups, with 6 mice in each group. The cells in each group were gently washed with PBS to remove excess medium and were then digested with 0.25% trypsin; the digestion was terminated with complete medium, and the cells were centrifuged, collected, and pelleted. The cells were added to an appropriate amount of normal saline and dispersed into single-cell suspensions for cell counting. Then, 3 × 10^6^ cells were resuspended in 50 μL physiological saline, mixed with 50 μL Matrigel Matrix (1:1), and inoculated into the armpits of nude mice. At 7, 14, 21, and 28 days after treatment, the mice were euthanized with 40 mg/kg pentobarbital (P3761, Sigma, St. Louis, USA), and tumors were harvested and analyzed. The short diameter (a) and long diameter (b) of each tumor were measured with Vernier calipers, and tumor volume was calculated according to the formula π (a^2^b)/6; the tumors were weighed with a balance. RT-qPCR and Western blot analysis were used to detect the protein expression of KDM4A, HIF1α, DDIT4, mTOR, phosphorylated mTOR, 4EBP1, phosphorylated 4EBP1, Ki67, cyclin D1, MMP-2, MMP-9, Bax, and Bcl-2 in tumor tissues.

### Statistical analysis

Statistical analysis was performed using Statistic Package for Social Science (SPSS) 21.0 software (IBM Corp. Armonk, NY, USA). The measurement data are expressed as the mean ± standard deviation. If the data followed a normal distribution and showed homogeneity of variance, matched data between two groups were compared using a paired t test. Comparisons among multiple groups were conducted by one-way analysis of variance (ANOVA) or at different time points by two-way ANOVA or repeated-measures ANOVA, followed by Tukey’s post hoc test or Bonferroni’s post hoc test. The Wilcoxon rank-sum (nonparametric) test was used for data with a skewed distribution and unequal variances. The enumeration data were analyzed by the chi-square test. Spearman and Pearson analyses were performed to assess correlations between indexes. A value of *p* < 0.05 indicated a significant difference.

## Results

### KDM4A is overexpressed in NPC clinical samples and cell lines

**Fig. 1:**
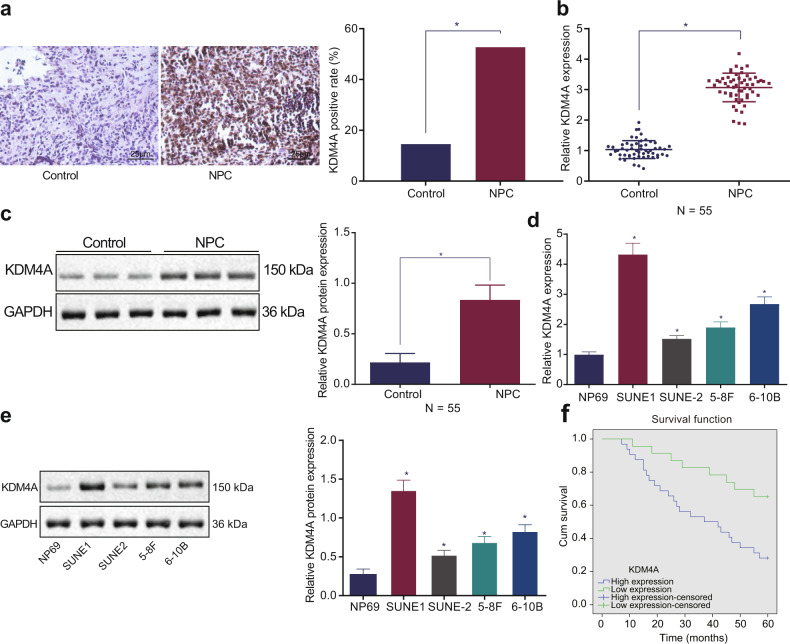
KDM4A is highly expressed in NPC clinical samples and cell lines, and this upregulation is associated with a poor prognosis in patients. **a** Immunohistochemical detection of KDM4A expression in clinical NPC tissues and adjacent tissues (×400, scale bar = 25 µm). **b** Detection of KDM4A mRNA expression in clinical NPC tissues and adjacent tissues by qRT-PCR. **c** Western blot analysis of KDM4A protein expression in clinical NPC tissues and adjacent tissues. **d** Screening for cell lines with the highest KDM4A mRNA expression by RT-qPCR. **e** Screening for cell lines with the highest KDM4A protein expression by Western blot analysis. **f** Kaplan–Meier method for survival analysis. *p < 0.05 vs. adjacent tissues or NP69 cells. The measurement data are expressed as the mean ± standard deviation. The data for cancer tissues and adjacent tissues were compared using paired *t* tests. One-way ANOVA was used for multigroup comparisons, and Kaplan-Meier analysis was used for survival analysis. The positive rate was expressed as a percentage and analyzed by the chi-square test. For patients, *n* = 55. The experiment was repeated three times independently.

First, to investigate the role of KDM4A in NPC, the expression of KDM4A was measured. Immunohistochemistry analysis revealed that the positive expression of KDM4A was indicated by brown-yellow staining, and KDM4A was mainly expressed in the nucleus but was also expressed in the cytoplasm. The expression of KDM4A in NPC tissues was much higher than that in adjacent tissues (Fig. 1a). RT-qPCR and Western blot analysis revealed that the mRNA (Fig. 1b) and protein (Fig. 1c) expression levels of KDM4A were much higher in NPC tissues than in adjacent tissues. Furthermore, RT-qPCR and Western blot analysis showed that compared with the NP69 cell line, KDM4A was expressed at a higher level in four NPC cell lines (SUNE1, SUNE-2, 5–8 F and 6–10B); of these cell lines, SUNE1 cells exhibited the highest KDM4A expression and were thus selected for subsequent experiments (Fig. 1d, e). The correlations between KDM4A and clinicopathological characteristics in 55 NPC cases were analyzed; the number of NPC clinical specimens with KDM4A high expression was far greater than the number of specimens with KDM4A low expression. Additionally, the expression of KDM4A was not related to patient sex, patient age, or tumor histological type (*p* > 0.05). In the tumor-node-metastasis (TNM) staging system, T represents the range of the primary tumor, which is expressed as T1 - T4. N indicates the existence and range of regional lymph node metastasis. When there is no lymph node involvement, the N stage is N0. N1-N3 indicates an increased degree and range of lymph node involvement. KDM4A expression was found to be related to the T stage, N stage, and clinical stage of tumors in the patients (Supplementary Table 2). Kaplan-Meier analysis revealed that the OS of patients with low KDM4A expression was much higher than that of patients with high KDM4A expression (Fig. 1f). Therefore, KDM4A was abnormally highly expressed in NPC tissues and cells, and high expression of KDM4A was associated with poor survival.

### KDM4A promotes HIF1α expression through its demethylase function

**Fig. 2:**
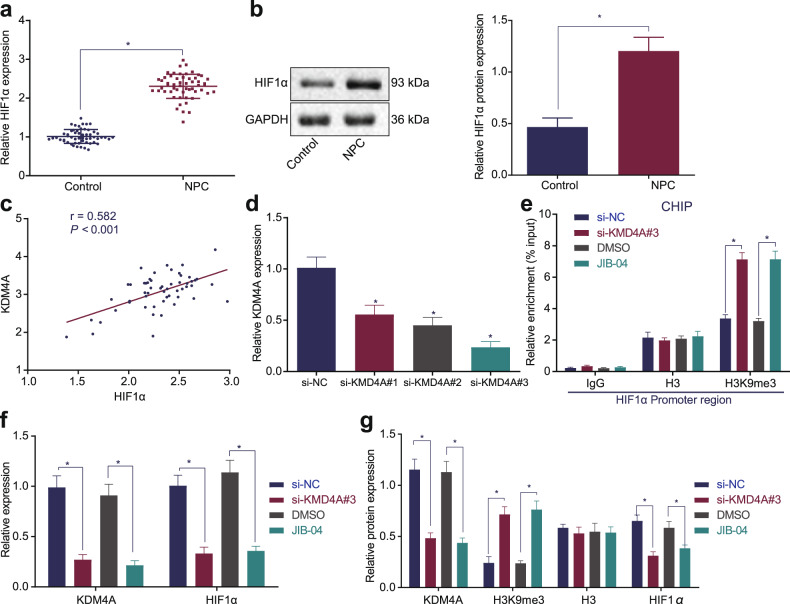
KDM4A increases HIF1α expression through its demethylase function. **a** RT-qPCR detection of HIF1α mRNA expression in clinical NPC tissues and adjacent tissues. **b** Western blot analysis of HIF1α protein expression in clinical NPC tissues and adjacent tissues. **c** Pearson correlation analysis of HIF1α mRNA expression with KDM4A mRNA expression. **d** Detection of the efficiency of KDM4A mRNA silencing by RT-qPCR. **e** ChIP detection of H3K9me3 enrichment in the HIF1α promoter region in each group of cells. **f** RT-qPCR detection of KDM4A and HIF1α mRNA expression in each group of cells. **g** Western blot analysis of KDM4A, H3K9me3, and HIF1α protein expression in each group of cells. *p < 0.05 vs. control cells or cells treated with si-NC or cells treated with DMSO. The measurement data are expressed as the mean ± standard deviation. Data between cancer tissues and adjacent tissues were compared using paired *t* tests. One-way ANOVA was used for multigroup comparisons. For patients, *n* = 55. The experiment was repeated three times independently.

Under the conditions of depletion or inactivation of histone demethylase KDM4A, H3K9me3 accumulates at the HIF1α site, resulting in reduced HIF1α mRNA expression and stability^9^. Additionally, other prior studies indicated that HIF1α was highly expressed in NPC and promoted the occurrence of NPC^13,17–19^. We speculated that KDM4A could promote the expression of HIF1α through its demethylase function. After RT-qPCR and Western blot analysis, we found that the expression of HIF1α was much higher in NPC tissues than in adjacent tissues (Fig. 2a, b). Pearson correlation analysis demonstrated that there was a significant positive correlation between the mRNA expression levels of HIF1α and KDM4A (*p* < 0.001; Fig. 2c). Therefore, we speculated that KDM4A could promote the expression of HIF1α. To test this hypothesis, three silencing sequences (si-KDM4A#1, si-KDM4A#2 and si-KDM4A#3) were designed, and their silencing efficiency was detected by RT-qPCR. The results showed that cells transfected with si-KDM4A#3 had the lowest KDM4A expression (Fig. 2d), so si-KDM4A#3 was therefore selected for subsequent experiments. SUNE1 cells were transfected with si-NC or si-KDM4A plasmids alone or combined with DMSO or JIB-04 (an inhibitor of KDM4A) were added. The ChIP results revealed that H3K9me3 enrichment in the HIF1α promoter region was increased in the cells treated with si-KDM4A or JIB-04 (*p* < 0.05), while H3 enrichment showed no changes (*p* > 0.05; Fig. 2e, Supplementary Fig. 1a), indicating that HIF1α is regulated by KDM4A and H3K9me3. RT-qPCR and Western blot analysis showed that KDM4A and HIF1α levels were decreased, while H3K9me3 levels were significantly increased (*p* < 0.05) and H3 levels were unaffected (*p* > 0.05; Fig. 2f, g, Supplementary Fig. 1b, c) in cells treated with si-KDM4A or JIB-04. In summary, KDM4A inhibited the enrichment of H3K9me3 in the HIF1α promoter region and thus inhibited the methylation of HIF1α to promote HIF1α expression.

### Silencing KDM4A could increase the H3K9me3 level to promote HIF1α methylation to inhibit NPC development

**Fig. 3:**
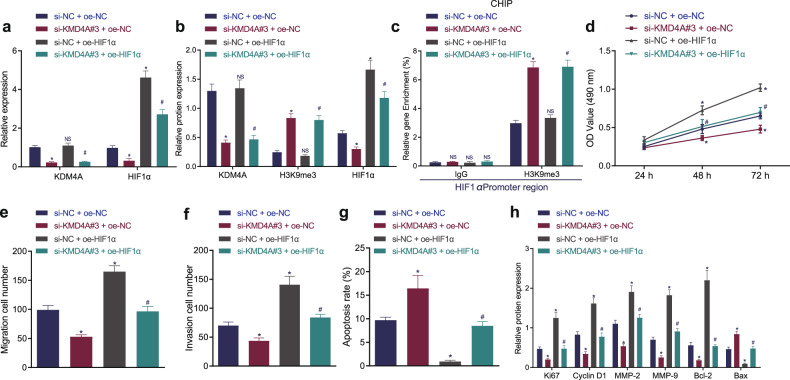
Silencing KDM4A increases the H3K9me3 level to enhance HIF1α methylation to inhibit NPC cell proliferation, migration, and invasion and induce apoptosis. **a**, RT-qPCR detection of KDM4A and HIF1α mRNA expression in each group of cells. **b**, Western blot analysis of KDM4A, H3K9me3, and HIF1α protein expression in each group of cells. **c**, ChIP detection of the enrichment of H3K9me3 in the HIF1α promoter region in each group of cells. **d**, MTT assay detection of cell proliferation in each group. **e**, Transwell assay detection of the cell migration ability of each group. **f**, Transwell assay for cell invasion ability. **g**, Flow cytometry detection of apoptosis in each group. **h**, Western blot analysis of the expression of cell proliferation (Ki67, cyclin D1), migration (MMP-2, MMP-9), and apoptosis (Bcl-2, Bax)-related proteins in each group. *p < 0.05 vs. cells treated with si-NC + OE-NC, # *p* < 0.05 vs. cells treated with si-NC + OE-HIF1α. NS meant no significant difference. The measurement data are expressed as the mean ± standard deviation. One-way ANOVA was used for multigroup comparisons, and cell viability at different time points was compared by two-way ANOVA. The experiment was repeated three times independently.

We then focused on the effect of the KDM4A/H3K9me3/HIF1α axis on the proliferation, migration, invasion and apoptosis of NPC cells. SUNE1 cells were cotransfected with si-KDM4A and OE-HIF1α. RT-qPCR and Western blot analysis revealed that the expression of KDM4A and HIF1α was decreased while the expression of H3K9me3 was increased by KDM4A silencing (*p* < 0.05), while overexpression of HIF1α did not affect the expression of KDM4A and H3K9me3 (*p* > 0.05) but increased HIF1α expression (*p* < 0.05). Moreover, compared with that in the cells treated with si-NC + OE-NC, KDM4A expression was reduced, while H3K9me3 and HIF1α expression was enhanced, in cells treated with si-KDM4A + OE-HIF1α. Compared with that after si-NC + OE-HIF1α treatment, KDM4A and HIF1α expression was decreased and H3K9me3 expression was increased after si-KDM4A + OE-HIF1α treatment (Fig. 3a, b, Supplementary Fig. 2a, b). The ChIP results showed that silencing KDM4A promoted the enrichment of H3K9me3 in the HIF1α promoter region (Fig. 3c, Supplementary Fig. 2c). MTT assay (Fig. 3d, Supplementary Fig. 2d), Transwell assay (Fig. 3e, f, Supplementary Fig. 2e, f), and flow cytometry (Fig. 3g, Supplementary Fig. 2g) depicted that the proliferation, invasion, and migration of SUNE1 cells were reduced, and apoptosis was enhanced by silencing KDM4A, but the opposite trend was observed after overexpressing HIF1α, which was reversed by cotreatment with si-KDM4A and OE-HIF1α. Finally, Western blot analysis was used to analyze the protein expression of proliferation (Ki67, cyclin D1), migration (MMP-2, MMP-9), and apoptosis (Bcl-2, Bax)-related factors. The results showed that silencing KDM4A reduced the protein expression of Ki67, cyclin D1, MMP-2, MMP-9, and Bcl-2 but increased Bax protein expression; overexpression of HIF1α caused the opposite trends, and these effects were all reversed by dual transfection with si-KDM4A and OE-HIF1α (Fig. 3h, Supplementary Fig. 2h). Therefore, silencing KDM4A increased the promoter methylation of HIF1α and downregulated HIF1α expression by increasing the H3K9me3 level, which inhibited NPC cell proliferation, migration, and invasion and promoted apoptosis.

### HIF1α targeted and upregulated DDIT4 to promote NPC development

**Fig. 4:**
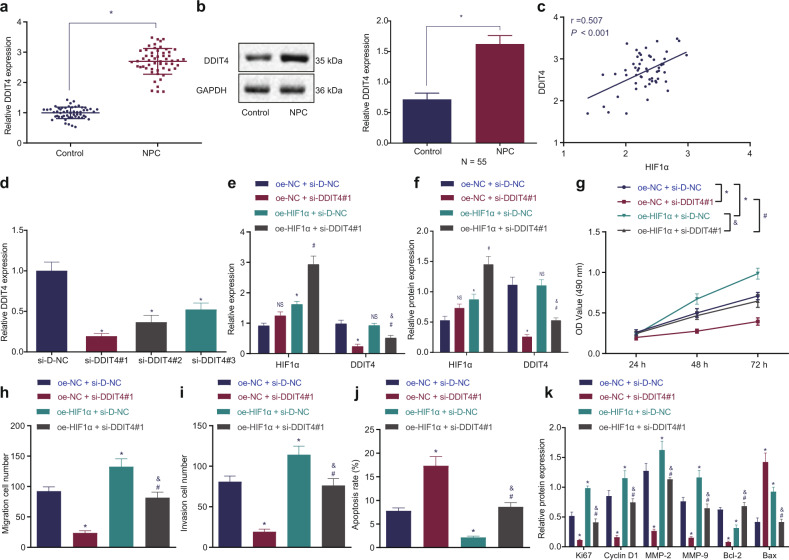
HIF1α promotes DDIT4 expression to stimulate cell proliferation, invasion, and migration and inhibit apoptosis in NPC. **a** RT-qPCR detection of DDIT4 mRNA expression in clinical NPC tissues and adjacent tissues. **b** Western blot analysis of DDIT4 protein expression in clinical NPC tissues and adjacent tissues. **c** Pearson analysis of the correlation between the expression of HIF1α and DDIT4. **d** RT-qPCR detection of DDIT4 mRNA silencing interference efficiency. **e** RT-qPCR detection of mRNA expression of HIF1α and DDIT4 in each group of cells. **f** Western blot analysis of HIF1α and DDIT4 protein expression in each group of cells. **g** MTT detection of cell proliferation in each group. **h** Transwell detection of cell migration ability in each group. **i** Transwell assay detection of the cell invasion ability of each group. **j** Flow cytometry detection of the apoptosis in each group. **k** Western blot analysis of the expression of cell proliferation (Ki67, cyclin D1), migration (MMP-2, MMP-9), and apoptosis (Bcl-2, Bax)-related factors in each group. *p < 0.05 vs. control cells, cells treated with si-D-NC or cells cotreated with OE-NC and si-D-NC, # *p* < 0.05 vs. cells cotreated with OE-NC and si-DDIT4. & *p* < 0.05 vs. cells cotreated with OE-HIF1α and si-D-NC. NS meant no significant difference. The measurement data are expressed as the mean ± standard deviation. Data between cancer tissues and adjacent tissues were compared using paired *t* tests. One-way ANOVA was used for multigroup comparisons. Cell viability at different time points was analyzed using two-way ANOVA. For patients, n = 55. The experiment was repeated three times independently.

DDIT4 has been documented to be a downstream target gene of HIF1α (a transcriptional activator), and its expression can be activated by HIF1α^14^. We speculated that HIF1α, which is overexpressed in NPC, could promote the expression of DDIT4. The results of RT-qPCR and Western blot analysis revealed that DDIT4 expression was much higher in NPC tissues than in adjacent tissues (Fig. 4a, b). Pearson correlation analysis showed a significant positive correlation between the expression of HIF1α and DDIT4 (*p* < 0.001; Fig. 4c). DDIT4 was positively correlated with HIF1α and was expressed at high levels in NPC.

Furthermore, we studied the impact of HIF1α on the development of NPC via DDIT4. First, three silencing sequences (si-DDIT4#1, si-DDIT4#2 and si-DDIT4#3) were designed, and RT-qPCR was used to detect their silencing efficiency. As shown in Fig. 4d, DDIT4 expression was the lowest in the presence of si-DDIT4#1, so this siRNA was selected for subsequent experiments. SUNE1 cells were treated with si-DDIT4 and OE-HIF1α. The results of RT-qPCR and Western blot analysis showed that compared with the OE-NC + si-D-NC transfection, there was no difference in HIF1α expression after transfection with OE-NC + si-DDIT4 (*p* > 0.05), and the expression of DDIT4 was decreased (*p* < 0.05); moreover, treatment with OE-HIF1α + si-D-NC caused no alterations in the DDIT4 expression (*p* > 0.05) but increased HIF1α expression (*p* < 0.05); HIF1α expression was increased and DDIT4 expression was decreased by treatment with OE-HIF1α + si-DDIT4 (*p* < 0.05). Compared with that with OE-NC + si-DDIT4 transfection, HIF1α and DDIT4 expression was increased with OE-HIF1α and si-DDIT4 transfection, while transfection with OE-HIF1α + si-DDIT4 reduced DDIT4 expression (*p* < 0.05; Fig. 4e, f, Supplementary Fig. 3a, b). As revealed in Fig. 4g-j and Supplementary Fig. 3c-f, silencing DDIT4 decreased SUNE1 cell proliferation, migration, and invasion but increased apoptosis, which was neutralized by transfection with OE-HIF1α alone or in combination with si-DDIT4. However, cotransfection with OE-HIF1α and si-DDIT4 inhibited SUNE1 cell proliferation, migration, and invasion but augmented apoptosis compared with OE-HIF1α transfection alone. Finally, Western blot analysis revealed that the protein expression of Ki67, cyclin D1, MMP-2, MMP-9, and Bcl-2 was decreased and that of Bax was increased after silencing DDIT4, and these changes were reversed by transfection with OE-HIF1α alone or combined with si-DDIT4. Additionally, the expression of Ki67, cyclin D1, MMP-2, MMP-9, and Bcl-2 proteins was lower, while that of Bax protein was higher, after cotransfection with OE-HIF1α and si-DDIT4 than after transfection with OE-HIF1α alone (Fig. 4k, Supplementary Fig. 3g). Thus, HIF1α activates its downstream gene DDIT4 to promote the proliferation, migration, and invasion and inhibit the apoptosis of NPC cells.

### DDIT4 activated the mTOR signaling pathway to promote NPC development

**Fig. 5:**
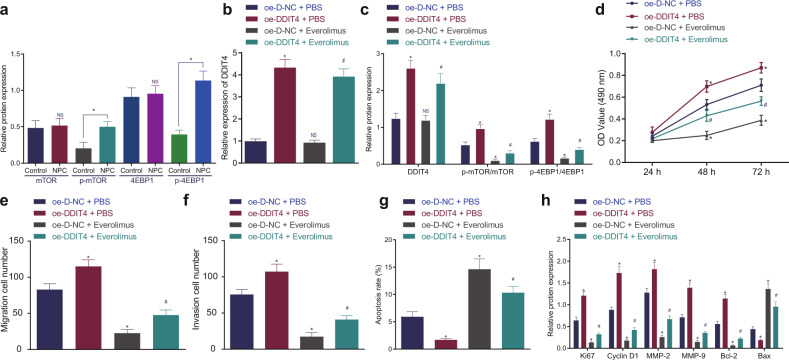
DDIT4 activates the mTOR signaling pathway to promote the proliferation, migration, and invasion and inhibit the apoptosis of NPC cells. **a** Western blot analysis of mTOR, phosphorylated mTOR, 4EBP1, and phosphorylated 4EBP1 protein expression in clinical NPC tissues and adjacent tissues. **b** RT-qPCR detection of DDIT4 mRNA expression in each group of cells. **c** Western blot analysis of the protein expression of DDIT4, mTOR, phosphorylated mTOR, 4EBP1, and phosphorylated 4EBP1 in each group of cells. **d** Detection of cell proliferation in each group by MTT assay. **e** Detection of cell migration ability in each group by Transwell assay. **f** Detection of the cell invasion ability of each group by Transwell assay. **g** Detection of apoptosis in each group by flow cytometry. **h** Analysis of the protein expression of cell proliferation (Ki67, cyclin D1), migration (MMP-2, MMP-9), and apoptosis (Bcl-2, Bax)-related factors in each group by Western blot analysis. *p < 0.05 vs. control cells or cells cotreated with OE-D-NC and PBS, # *p* < 0.05 vs. cells cotreated with OE-D-NC and everolimus. NS meant no significant difference. The measurement data are expressed as the mean ± standard deviation. Data between cancer tissues and adjacent tissues were compared using paired *t* tests. One-way ANOVA was used for multigroup comparisons, and cell viability at different time points was compared using two-way ANOVA. For patients, *n* = 55. The experiment was repeated three times independently.

Accumulating evidence has indicated that DDIT4 can activate the mTOR signaling pathway, which promotes the occurrence of NPC^14,20^. Thus, we first analyzed the clinical NPC tissues and adjacent tissues by Western blot analysis. The results showed that the expression of phosphorylated mTOR and phosphorylated 4EBP1 was enhanced in NPC tissues compared to adjacent tissues (*p* < 0.05), while 4EBP1 and mTOR expression was not significantly different (*p* > 0.05; Fig. 5a). Furthermore, SUNE1 cells were treated with both OE-DDIT4 and the mTOR pathway inhibitor everolimus. From the results of RT-qPCR and Western blot analysis, compared with treatment with OE-D-NC + PBS, the expression of DDIT4, phosphorylated mTOR, and phosphorylated 4EBP1 was increased with OE-DDIT4 + PBS treatment (*p* < 0.05). There was no difference in DDIT4 expression after OE-D-NC + everolimus treatment (*p* > 0.05), and the expression of phosphorylated mTOR and phosphorylated 4EBP1 was decreased (*p* < 0.05); DDIT4 expression was elevated and phosphorylated mTOR and phosphorylated 4EBP1 expression was diminished following OE-DDIT4 + PBS treatment (*p* < 0.05). Compared with that in cells treated with OE-D-NC + everolimus, the expression of DDIT4, phosphorylated mTOR, and phosphorylated 4EBP1 was increased in cells treated with OE-DDIT4 + everolimus (*p* < 0.05). However, there was no significant difference in mTOR and 4EBP1 expression among the groups (*p* > 0.05; Fig. 5b, c). Based on the MTT assay (Fig. 5d), Transwell assay (Fig. 5e, f), and flow cytometry (Fig. 5g), SUNE1 cell proliferation, invasion, and migration were promoted, while apoptosis was inhibited in DDIT4-overexpressing cells. However, everolimus treatment resulted in the inhibition of SUNE1 cell proliferation, invasion, and migration and the promotion of apoptosis, which was abrogated by cotreatment with OE-DDIT4 and everolimus (*p* < 0.05). Moreover, Western blot analysis showed that in the presence or absence of everolimus, Ki67, cyclin D1, MMP-2, MMP-9, and Bcl-2 protein expression was increased, while Bax protein expression decreased with the overexpression of DDIT4 (*p* < 0.05; Fig. 5h). In summary, the mTOR signaling pathway is activated in NPC, and DDIT4 overexpression promotes the proliferation, migration, invasion and inhibits the apoptosis of NPC cells by partially activating the mTOR signaling pathway.

### KDM4A promoted NPC development by promoting the HIF1α/DDIT4/mTOR axis

**Fig. 6:**
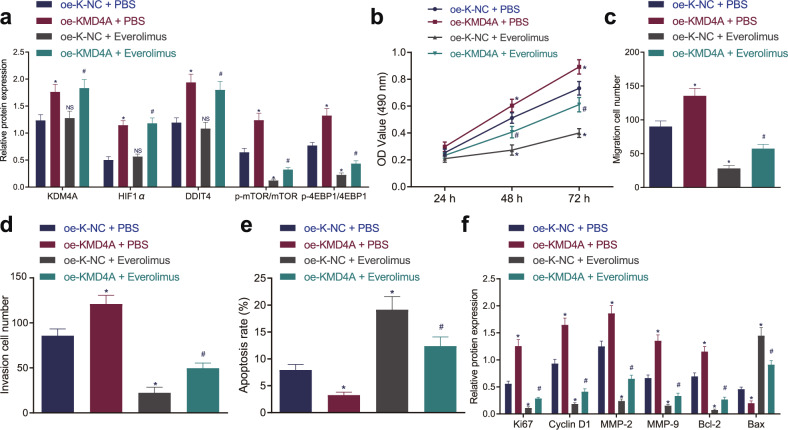
KDM4A promotes the proliferation, migration and invasion and inhibits the apoptosis of NPC cells by activating the HIF1α/DDIT4/mTOR axis. **a** Western blot analysis of KDM4A, HIF1α, DDIT4, mTOR, phosphorylated mTOR, 4EBP1, and phosphorylated 4EBP1 protein expression in each group of cells. **b** Detection of cell proliferation in each group by MTT assay. **c** Detection of cell migration ability in each group by Transwell assay. **d** Detection of the cell invasion ability of each group by Transwell assay. **e** Cell apoptosis detected by flow cytometry. **f** Analysis of the protein expression of cell proliferation (Ki67, cyclin D1), migration (MMP-2, MMP-9), and apoptosis (Bcl-2, Bax)-related factors in each group by Western blot analysis. *p < 0.05 vs. cells cotreated with OE-K-NC and PBS, # *p* < 0.05 vs. cells cotreated with OE-K-NC and everolimus. NS meant no significant difference. The measurement data are expressed as the mean ± standard deviation. One-way ANOVA was used for multigroup comparisons, and cell viability at different time points was compared using two-way ANOVA. The experiment was repeated three times independently.

To study the effect of the KDM4A/HIF1α/DDIT4/mTOR axis on NPC development, SUNE1 cells were treated with OE-KDM4A and treated with the mTOR pathway inhibitor everolimus. The results of Western blot analysis showed that compared with cells treated with OE-K-NC (NC for OE-KDM4A) + PBS, the expression of KDM4A, HIF1α, DDIT4, phosphorylated mTOR, and phosphorylated 4EBP1 was increased in cells treated with OE-KDM4A + PBS (*p* < 0.05); there was no difference in KDM4A, HIF1α, and DDIT4 expression in cells treated with OE-K-NC + everolimus (*p* > 0.05), but the expression of phosphorylated mTOR and phosphorylated 4EBP1 was decreased (*p* < 0.05); the expression of KDM4A, HIF1α, and DDIT4 was increased while that of phosphorylated mTOR and phosphorylated 4EBP1 was decreased in cells treated with OE-KDM4A + everolimus (*p* < 0.05). Compared with that in cells treated with OE-K-NC + everolimus, the expression of KDM4A, HIF1α, DDIT4, phosphorylated mTOR, and phosphorylated 4EBP1 was increased in cells treated with OE-KDM4A + everolimus (*p* < 0.05). There was no significant difference in mTOR and 4EBP1 expression among the groups (*p* > 0.05; Fig. 6a). As depicted in Fig. 6b-e, the proliferation, invasion, and migration of SUNE1 cells was enhanced in KDM4A-overexpressing cells, but apoptosis was reduced. However, everolimus treatment diminished the proliferation, invasion, and migration of SUNE1 cells and increased apoptosis, which was rescued by overexpressing KDM4A. Additionally, as indicated by the results of Western blot analysis, upregulation of Ki67, cyclin D1, MMP-2, MMP-9, Bcl-2 protein expression and downregulation of Bax protein expression were observed after KDM4A overexpression, and these effects the opposite of those after everolimus treatment. Cotreatment with OE-KDM4A + everolimus reversed the effects of everolimus treatment (*p* < 0.05; Fig. 6f). Therefore, highly expressed KDM4A partially activates the HIF1α/DDIT4/mTOR axis to promote the proliferation, migration, and invasion and inhibit the apoptosis of NPC cells.

### Knockdown of KDM4A inactivated the mTOR signaling pathway by inhibiting the HIF1α/DDIT4 axis to suppress tumor growth in a nude mouse xenotransplant model

**Fig. 7:**
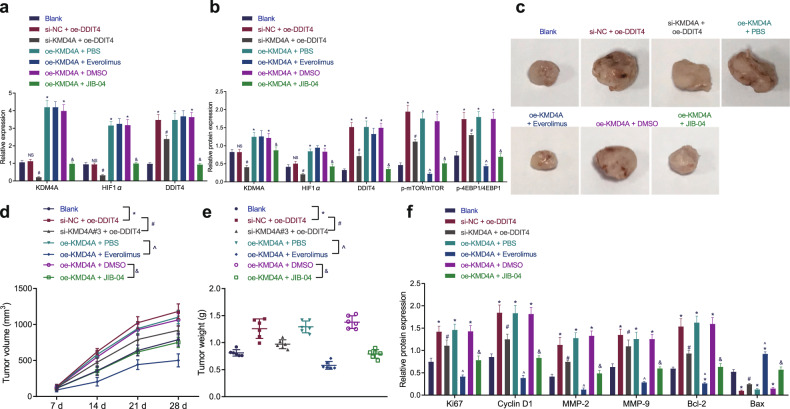
KDM4A silencing inhibits the HIF1α/DDIT4 axis to inactivate the mTOR signaling pathway, thus attenuating the growth of transplanted tumors in nude mice. **a** Detection of KDM4A, HIF1α, and DDIT4 mRNA expression in nude mouse tumor tissues by RT-qPCR. **b** Analysis of the expression of KDM4A, HIF1α, DDIT4, mTOR, phosphorylated mTOR, 4EBP1, and phosphorylated 4EBP1 in nude mouse tumor tissues of each group by Western blot analysis. **c** Anatomy of tumors in nude mice in each group. **d** Line graph of tumor volume changes in nude mice in each group. **e** Tumor weight comparison of nude mice in each group. **f** Analysis of the protein expression of proliferation (Ki67, cyclin D1), migration (MMP-2, MMP-9), and apoptosis (Bcl-2, Bax)-related factors in nude mouse tumor tissues of each group by Western blot analysis. *p < 0.05 vs. the blank group, # *p* < 0.05 vs. mice cotreated with si-NC and OE-DDIT4, & *p* < 0.05 vs. mice cotreated with OE-KDM4A and DMSO, ^ *p* < 0.05 vs. mice cotreated with OE-KDM4A and PBS. NS meant no significant difference. The measurement data are expressed as the mean ± standard deviation. One-way ANOVA was used for multigroup comparisons, and tumor volume at different time points was compared using repeated-measures ANOVA. For mice, *n* = 6 per group.

We performed in vivo experiments to study the effect of the KDM4A/HIF1α/DDIT4/mTOR axis on the tumorigenesis of NPC cells. According to the experimental design requirements, 5 groups of stably transfected SUNE1 cells were injected into the underarms of nude mice to construct nude mouse tumor transplantation models. The results of RT-qPCR and Western blot analysis showed that compared with that in mice in the blank group, the expression of KDM4A and HIF1α was not different in the mice in the si-NC + OE-DDIT4 group (*p* > 0.05), while that of DDIT4, phosphorylated mTOR, and phosphorylated 4EBP1 was increased (*p* < 0.05). In mice in the si-KDM4A + OE-DDIT4 group, the expression of KDM4A and HIF1α was decreased, while that of DDIT4, phosphorylated mTOR, and phosphorylated 4EBP1 was increased (*p* < 0.05). In mice in the OE-KDM4A + PBS group, KDM4A, HIF1α, DDIT4, phosphorylated mTOR, and phosphorylated 4EBP1 expression was increased (*p* < 0.05). In mice in the OE-KDM4A + everolimus, the expression of KDM4A, HIF1α, and DDIT4 was increased, while that of phosphorylated mTOR and phosphorylated 4EBP1 was decreased (*p* < 0.05). Mice in the OE-KDM4A + DMSO group showed upregulated expression of KDM4A, HIF1α, DDIT4, phosphorylated mTOR and phosphorylated 4EBP1 (*p* < 0.05), but there was no difference in these in the OE-KDM4A + JIB-04 group versus the control group (*p* > 0.05). Compared with that in the mice in the si-NC + OE-DDIT4 group, the expression of KDM4A, HIF1α, DDIT4, phosphorylated mTOR, and phosphorylated 4EBP1 was reduced in the mice in the si-KDM4A + OE-DDIT4 group (*p* < 0.05). Compared with the mice in the OE-KDM4A + PBS group, the expression of KDM4A, HIF1α, and DDIT4 was not different in the mice in the OE-KDM4A and everolimus group (*p* > 0.05), while that of phosphorylated mTOR and phosphorylated 4EBP1 was reduced (*p* < 0.05). 4EBP1 and mTOR expression was not significantly different among the groups (*p* > 0.05). In comparison to the mice in the OE-KDM4A + DMSO group, mice in the OE-KDM4A + JIB-04 group showed decreased KDM4A, HIF1α and DDIT4 expression as well as decreased mTOR and 4EBP1 phosphorylation (*p* < 0.05; Fig. 7a, b, Supplementary Fig. 4a). These data suggest that knockdown of KDM4A prevents mTOR signaling pathway activation by inhibiting the HIF1α/DDIT4 axis.

Furthermore, the tumor growth curves of nude mice were generated over 4 consecutive weeks. Compared with that of mice in the blank group, tumor growth (Fig. 7c, d, Supplementary Fig. 4b) and weight (Fig. 7e, Supplementary Fig. 4c) were enhanced in the mice of the si-NC + OE-DDIT4, OE-KDM4A + PBS or OE-KDM4A + DMSO groups (*p* < 0.05), and this effect was neutralized by KDM4A silencing or everolimus treatment (*p* < 0.05). There were no alterations in the tumor growth or weight in the OE-KDM4A + JIB-04 group versus the blank group (*p* > 0.05). Additionally, tumor growth and weight were decreased in the OE-KDM4A + JIB-04 group mice compared to the OE-KDM4A + DMSO group mice (*p* < 0.05). In addition, Western blot analysis illustrated that the protein expression of Ki67, cyclin D1, MMP-2, MMP-9, and Bcl-2 was increased while that of Bax was decreased in the si-NC + OE-DDIT4, OE-KDM4A + PBS and OE-KDM4A + DMSO groups (*p* < 0.05). Nevertheless, these changes were attenuated by KDM4A silencing or everolimus treatment (*p* < 0.05). Additionally, compared with those in the OE-KDM4A + DMSO group, mice in the OE-KDM4A + JIB-04 group showed downregulated expression of Ki67, cyclin D1, MMP-2, MMP-9, and Bcl-2 and upregulated expression of Bax (*p* < 0.05; Fig. 7f, Supplementary Fig. 4d). Therefore, silencing KDM4A inhibits mTOR signaling pathway activation by repressing the HIF1α/DDIT4 axis to suppress the growth of transplanted tumors in nude mice.

## Discussion

NPC is considered a rare cancer in most parts of the world, but it has high morbidity and mortality rates due to its early metastasis and chemotherapy toxicity^21,22^. Radiation therapy is the main treatment for NPC, but radiation resistance can prevent effective treatment^23^. KDM4A has been found to contribute to the Warburg effect in the NPC process^8^, but the specific mechanism of KDM4A in NPC remains poorly defined. Our present study provides evidence that KDM4A promotes the expression of HIF1α to upregulate DDIT4, which activates the mTOR signaling pathway, thus promoting NPC progression by increasing cell proliferation, migration, and invasion while inhibiting apoptosis.

The first finding of this study was that KDM4A is expressed at a high level in NPC cells, in which it promotes proliferation, migration, and invasion and inhibits apoptosis; these effects are accompanied by the upregulation of the Ki67, cyclin D1, MMP-2, MMP-9, and Bcl-2 protein expression and the downregulation of Bax protein expression. It is well known that histone methylation can dynamically regulate a variety of developmental and physiological processes^24^. A previous study reported that histone demethylases play a vital role in various biological processes, and their mode of action depends on their demethylase activity^25^. In line with our results, Su et al. observed that KDM4A was upregulated in NPC tissues and was positively correlated with tumor stage, metastasis and clinical stage and that KDM4A overexpression promoted NPC cell proliferation, invasion, and migration^8^. Ki67 is associated with prognosis in patients with NPC^26^. Cyclin D1, a key cell cycle regulator and a candidate proto-oncogene, plays a vital role in regulating the radiosensitivity and cell cycle distribution of NPC cells^27^. In addition, the increase in migration and invasion is caused by the upregulation of MMP-2 and MMP-9 expression and activity^28^. Bax is a proapoptotic protein from the Bcl-2 family that is at the core of apoptosis regulation^29^. It has been reported that Bax is downregulated in NPC tissues^30^. Our study also found that JIB-04 could increase the level of H3K9me3, which led to a decrease in the level of HIF1α. Moreover, we unveiled that KDM4A suppressed HIF1α methylation by inhibiting H3K9me3 enrichment in the HIF1α promoter region and promoted an increase in HIF1α expression. JIB-04 is a small molecule compound that can inhibit the demethylation activity of the KDM enzyme family^31^. It has been reported that JIB-04 can reduce the demethylation activity of KDM4A to increase the H3K36me3 level^32^. Additionally, JIB-04 can increase the H3K4me3 level by inhibiting the expression of the methyltransferase KDM5B, thereby increasing the sensitivity of cancer cells to radiotherapy^33^. One recent study also noted that JIB-04 can inhibit the demethylation function of the methyltransferase MINA53 to increase the H3K36me3 level^34^. Thus, we believe that JIB-04 could promote histone methylation by inhibiting the action of demethylase. Similarly, a prior study discovered that H3K9me3 could accumulate on the HIF1α locus after inactivation or depletion of KDM4A, thus reducing HIF1α mRNA expression and stability^9^. Thus, we speculated that KDM4A promotes NPC development by upregulating HIF1α expression. However, the specific relationship between the H3K9me3 level and HIF1α needs to be further explored.

In subsequent experiments, we found that HIF1α was highly expressed in NPC tissues and that it activated its downstream factor DDIT4 to promote the proliferation, migration, and invasion and inhibit the apoptosis of SUNE1 cells. In recent years, many studies have identified one or more transcription factors as driving forces for the development of biological or disease processes^11^. HIF1α transcriptionally regulates many key aspects of tumor development and progression by promoting a more aggressive tumor phenotype, which is characterized by increased proliferation and aggressiveness and neovascularization^18^. The transcription factor HIF1α may be a potential therapeutic target for NPC and other diseases^17^. Additionally, previous research demonstrated that oleuropein enhances the radiosensitivity of NPC by downregulating HIF1α^19^. DDIT4 modulates the fate of mesenchymal stem cells by mediating the interaction between HIF1α and the mTOR signaling pathway^14^. Based on these research findings and the present results, we contended that HIF1α enhanced NPC by upregulating DDIT4 expression.

The present study confirmed that DDIT4 is overexpressed in NPC, which activates the mTOR signaling pathway to promote NPC development. DDIT4 contributes to the proliferation and occurrence of gastric cancer^35^. DDIT4 mediates methamphetamine-induced autophagy and apoptosis through the mTOR signaling pathway^36^. Rapamycin inhibits the suppression of NPC stem cell characteristics through the mTOR signaling pathway^37^. Our data suggest that the mTOR signaling pathway enhances NPC progression.

In conclusion, this paper proposes that KDM4A upregulates DDIT4 to activate the mTOR signaling pathway by increasing the expression of the transcription factor HIF1α, thus enhancing NPC progression, which improves the understanding of the mechanisms of NPC. However, the current study only presents the theoretical basis for this mechanism in NPC. Therefore, clinical experiments with a fully developed KDM4A-based therapeutic agent are needed. Moreover, further clinical investigation of the mechanism should be performed with a more diverse study population to support a promising avenue for the treatment of NPC.

## Supplementary information

Supplementary Information
