# Supplementary material for: Histone demethylase KDM4A plays an oncogenic role in nasopharyngeal carcinoma by promoting cell migration and invasion
Source: Exp Mol Med. 2021 Aug 12;53(8):1207–17. doi: 10.1038/s12276-021-00657-0 (PMC8417295; doi:10.1038/s12276-021-00657-0)
Supplement: Supplementary file 1 — Supplementary Information [file 12276_2021_657_MOESM1_ESM.doc]

**
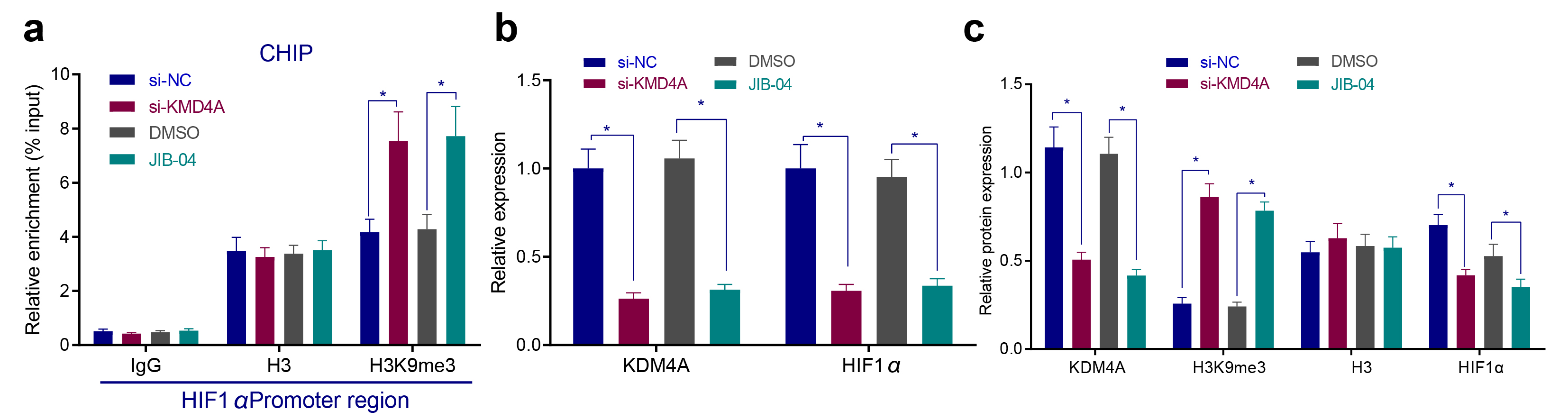
**

**Supplementary Figure 1** **KMD4A promotes HIF1α expression through its demethylase function. a, The enrichment of H3K9me3 in HIF1α promoter in each group was detected by ChIP. b, mRNA expression of KMD4A, H3K9me3 and HIF1α in cells by RT-qPCR. c, Protein expression of KMD4A, H3K9me3 and HIF1α in cells by Western blot analysis.** * *p* < 0.05 vs. cells treated with si-NC or DMSO. The measurement data were expressed as mean ± standard deviation.

**
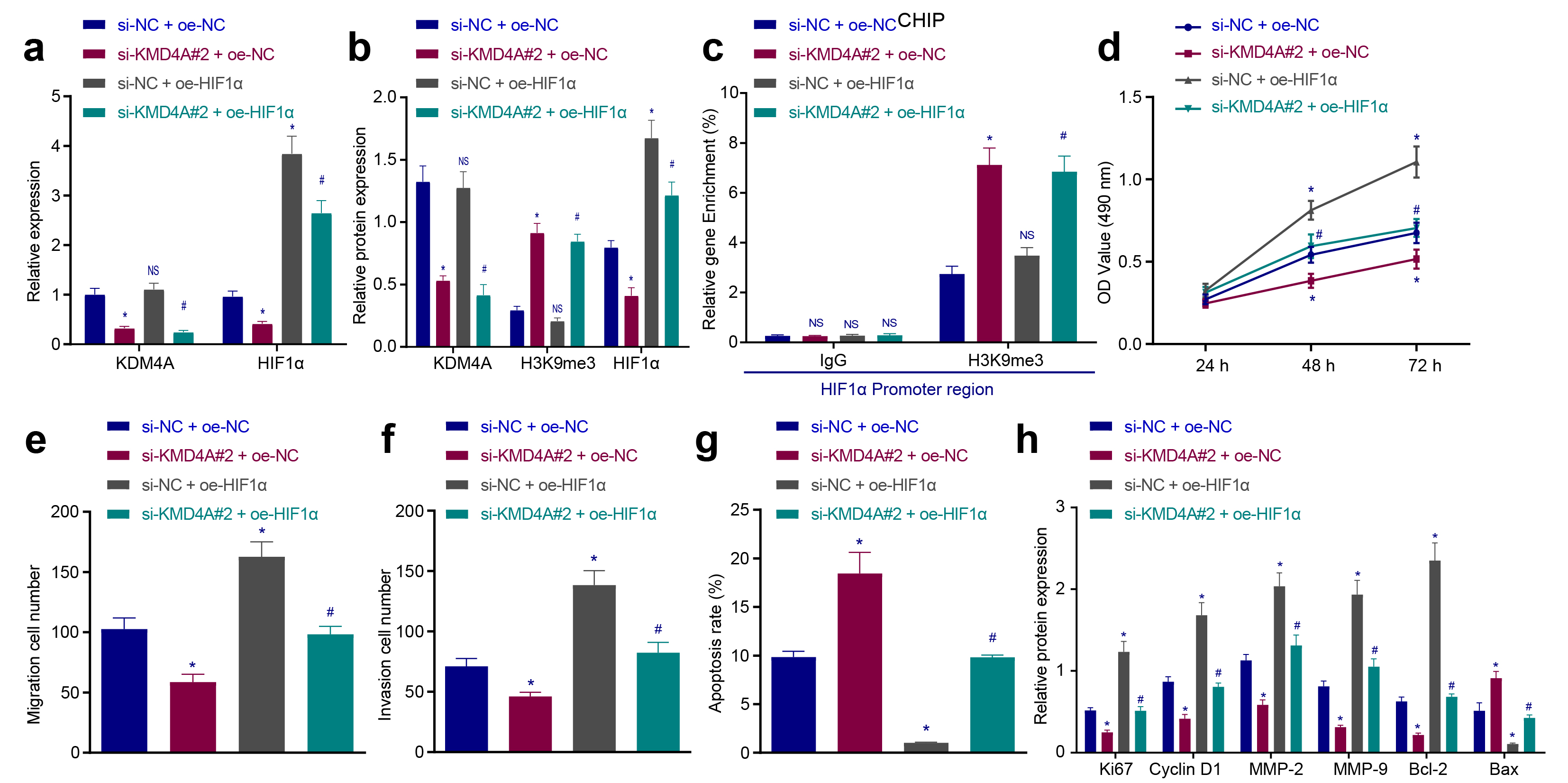
**

**Supplementary Figure 2 Silencing KDM4A recruits H3K9me3 to enhance HIF1α methylation to inhibit NPC cell proliferation, migration, invasion, and pro-apoptosis.** a, RT-qPCR detection of mRNA expression of KDM4A and HIF1α in each group of cells. b, Western blot analysis of KDM4A, H3K9me3, and HIF1α protein expression in each group of cells. c, ChIP detection the enrichment of H3K9me3 in the HIF1α promoter region in each group of cells. d, MTT assay detection the cell proliferation in each group. e, Transwell assay detection the cell migration ability of each group. f, Transwell assay for cell invasion ability. g, Flow cytometry detection of apoptosis in each group. h, Western blot analysis of expression of cell proliferation (Ki67, Cyclin D1), migration (MMP-2, MMP-9), and apoptosis (Bcl-2, Bax)-related proteins in each group. * *p* < 0.05 vs. cells treated with si-NC + oe-NC, # *p* < 0.05 vs. cells treated with si-NC + oe-HIF1α. NS meant no significant difference. The measurement data were expressed as mean ± standard deviation. One-way ANOVA was used for multi-group data comparison and cell viability at different time points was compared by two-way ANOVA. The experiment was repeated three times independently.

**
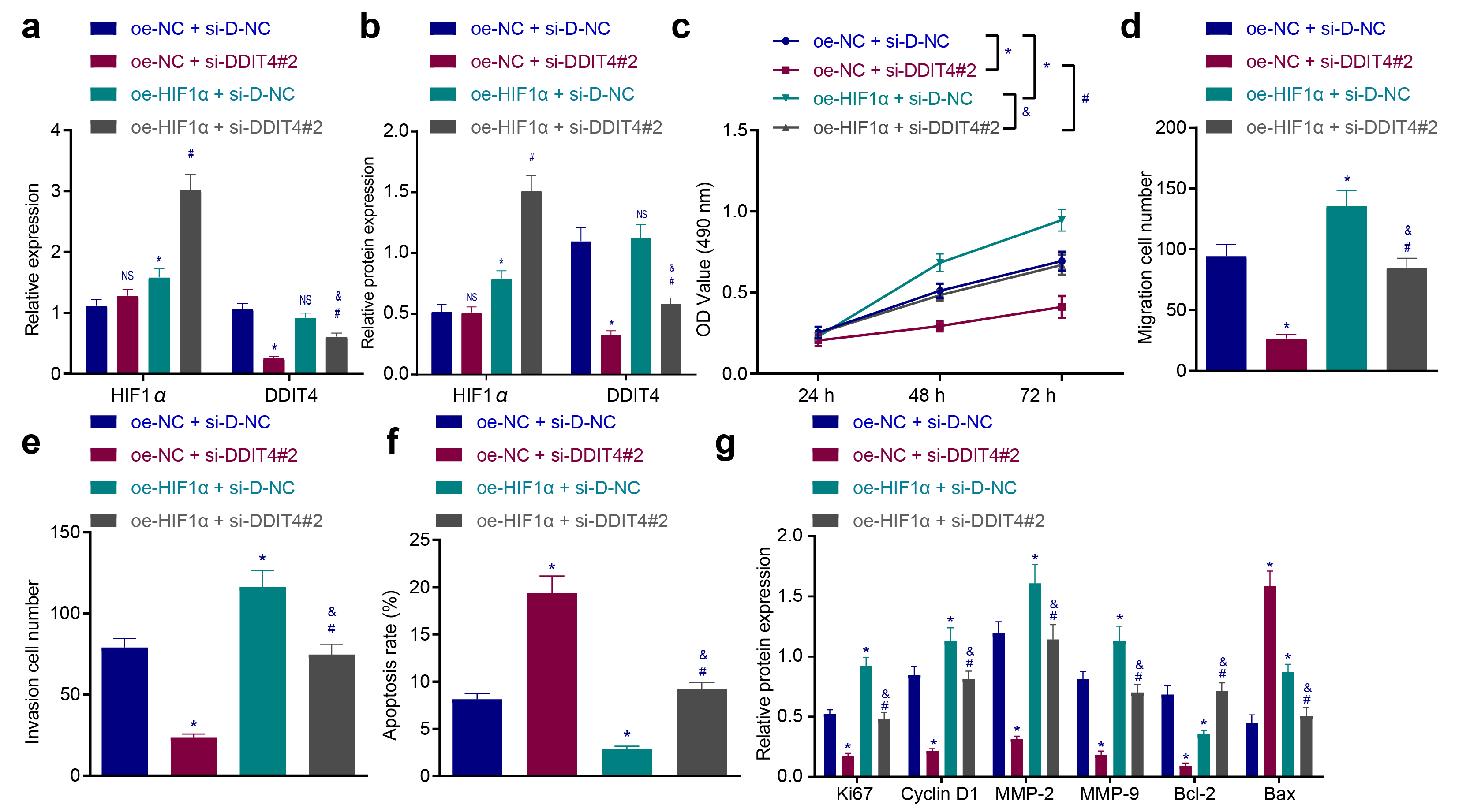
**

**Supplementary Figure 3 HIF1α promotes DDIT4 expression to stimulate cell proliferation, invasion, and migration but to reduce apoptosis in NPC.** a, RT-qPCR detection of mRNA expression of HIF1α and DDIT4 in each group of cells. b, Western blot analysis of HIF1α and DDIT4 protein expression in each group of cells. c, MTT detection of cell proliferation in each group. d, Transwell detection cell migration ability in each group. e, Transwell assay detection the cell invasion ability of each group. f, Flow cytometry detection the apoptosis of each group. g, Western blot analysis of the expression of cell proliferation (Ki67, Cyclin D1), migration (MMP-2, MMP-9), and apoptosis (Bcl-2, Bax)-related factors in each group. * *p* < 0.05 vs. control cells, cells treated with si-D-NC or cells co-treated with oe-NC and si-D-NC, # *p* < 0.05 vs. cells co-treated with oe-NC and si-DDIT4. & *p* < 0.05 vs. cells co-treated with oe-HIF1α and si-D-NC. NS meant no significant difference. The measurement data were expressed as mean ± standard deviation. Data between cancer tissues and adjacent tissues were compared using paired *t* test. One-way ANOVA was used for multi-group data comparison. Cell viability at different time points was used two-way ANOVA. For patients, n = 55. The experiment was repeated three times independently.

**
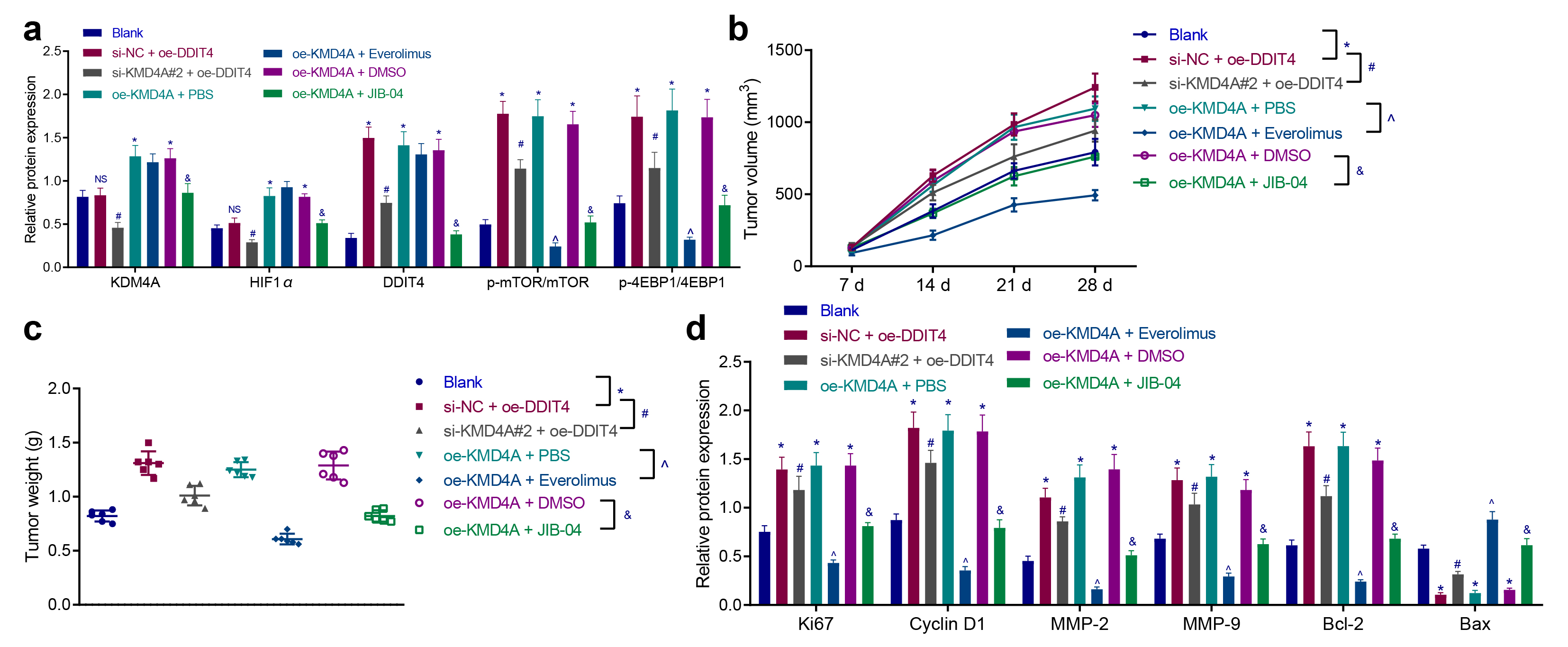
**

**Supplementary Figure 4 KDM4A silencing inhibits the HIF1α/DDIT4 axis to inactivate the mTOR signaling pathway, thus attenuating the growth of nude mice transplanted tumors.** a, Analysis the expression of KDM4A, HIF1α, DDIT4, mTOR, phosphorylated mTOR, 4EBP1, and phosphorylated 4EBP1 in tumor tissues of nude mice in each group by Western blot analysis. c, Line graph of tumor volume changes in nude mice in each group. c, Tumor weight comparison of nude mice in each group. d, Analysis of the protein expression of proliferation (Ki67, Cyclin D1), migration (MMP-2, MMP-9), and apoptosis (Bcl-2, Bax)-related factors in nude mice tumor tissues of each group by Western blot analysis. * *p* < 0.05 vs. blank group, # *p* < 0.05 vs. mice co-treated with si-NC and oe-DDIT4, ∧ *p* < 0.05 vs. mice co-treated with oe-KDM4A and PBS. NS meant no significant difference. The measurement data were expressed as mean ± standard deviation. One-way ANOVA was used for multi-group data comparison and tumor volume at different time points was compared using repeated measures ANOVA. For mice, n = 6 in each group.

**Supplementary Table 1** RT-qPCR primer sequences

| Primer | Sequence (5'-3') | |
| --- | --- | --- |
| KDM4A | F: ATCCCAGTGCTAGGATAATGACC | R: ACTCTTTTGGAGGAACCCTTG |
| HIF1α | F: CTCAAAGTCGGACAGCCTCA | R: CCCTGCAGTAGGTTTCTGCT |
| DDIT4 | F: GGACCAAGTGTGTTTGTTGTTTG | R: CACCCACCCCTTCCTACTCTT |
| GAPDH | F: TGACGCTGGGGCTGGCATTG | R: GCTCTTGCTGGGGCTGGTGG |

Note: KDM4A, lysine specific demethylase 4A; HIF1α, hypoxia-inducible factor-1α, DDIT4; DNA damage inducible transcript 4 gene; GAPDH, glyceraldehyde-3-phosphate dehydrogenase; RT-qPCR, reverse transcription quantitative polymerase chain reaction; F, forward; R, reverse.

**Supplementary Table 2 Relationship between KDM4A expression and clinicopathological features of NPC patients**

| Clinicopathological parameter | Case number (n = 55) | Expression of KDM4A | | *p* value |
| --- | --- | --- | --- | --- |
|  |  | Negative (%) | Positive (%) |  |
| Gender |  |  |  |  |
| male | 37 | 19 (51.35) | 18 (48.65) | 0.407 |
| female | 18 | 7 (38.89) | 11 (61.11) |  |
| Age |  |  |  |  |
| < 50 | 25 | 11 (44.00) | 14 (56.00) | 0.788 |
| ≥ 50 | 30 | 15 (50.00) | 15 (50.00) |  |
| Histological type |  |  |  |  |
| DNKC | 27 | 15 (56.56) | 12 (44.44) | 0.285 |
| UDC | 28 | 11 (39.29) | 17 (60.71) |  |
| T grading |  |  |  |  |
| T1 - T2 | 35 | 21 (60.00) | 14 (40.00) | 0.024 |
| T3 - T4 | 20 | 5 (25.00) | 75 (75.00) |  |
| N grading |  |  |  |  |
| N0 | 17 | 12 (70.59) | 5 (29.41) | 0.039 |
| N1 - N3 | 38 | 14 (36.84) | 24 (63.16) |  |
| Clinical stages |  |  |  |  |
| Ι - II | 33 | 20 (60.61) | 13 (39.39) | 0.027 |
| III - IV | 22 | 6 (27.27) | 16 (72.73) |  |

Notes: The result of this data was counted data, expressed by the number of cases and percentages, and analyzed by chi-square test. n = 55, and *p* < 0.05 indicated that the difference was statistically significant.
